# Conjugation genes are common throughout the genus *Rickettsia* and are transmitted horizontally

**DOI:** 10.1098/rspb.2009.0875

**Published:** 2009-07-16

**Authors:** Lucy A. Weinert, John J. Welch, Francis M. Jiggins

**Affiliations:** 1 Institute of Evolutionary Biology, University of Edinburgh, Edinburgh EH9 3JT, UK; 2 Department of Genetics, University of Cambridge, Cambridge CB2 3EH, UK

**Keywords:** plasmid, gene exchange, arthropod, *Wolbachia*, phylogeny

## Abstract

*Rickettsia* are endosymbionts of arthropods, some of which are vectored to vertebrates where they cause disease. Recently, it has been found that some *Rickettsia* strains harbour conjugative plasmids and others encode some conjugative machinery within the bacterial genome. We investigated the distribution of these conjugation genes in a phylogenetically diverse collection of *Rickettsia* isolated from arthropods. We found that these genes are common throughout the genus and, in stark contrast to other genes in the genome, conjugation genes are frequently horizontally transmitted between strains. There is no evidence to suggest that these genes are preferentially transferred between phylogenetically related strains, which is surprising given that closely related strains infect similar host species. In addition to detecting patterns of horizontal transmission between diverse *Rickettsia* species, these findings have implications for the evolution of pathogenicity, the evolution of *Rickettsia* genomes and the genetic manipulation of intracellular bacteria.

## 1. Introduction

*Rickettsia* are facultative endosymbionts of arthropods, many of which are known to be transmitted vertically from mother to offspring (reviewed in Perlman *et al*. 2006; Weinert *et al*. 2009). A small proportion of strains are also vectored to humans and other vertebrate hosts where they cause diseases such as typhus fever and Rocky Mountain spotted fever (Azad & Beard 1998). In addition, some strains have evolved adaptations to help them spread through arthropod populations, such as killing male hosts in order to redistribute resources to infected female siblings (Lawson *et al*. 2001; Weinert *et al*. 2007), or inducing parthenogenesis to make all of the infected hosts female (the sex that will transmit the bacterium; Hagimori *et al*. 2006).

Owing to their medical importance, the genomes of 14 strains of *Rickettsia* have been sequenced. These genomes are smaller than those of their free-living counterparts and contain a larger proportion of pseudogenes and non-coding DNA. This is indicative of genome degradation consistent with relaxed selection (owing to substitution of gene products by the host), ineffective selection (owing to small effective population size and limited recombination; Balbi *et al*. 2009) and a mutational bias towards deletions (Andersson & Andersson 2001). *Rickettsia* genomes also encode for less recombination machinery (Andersson *et al*. 1998), which is reflected in very low recombination rates between *Rickettsia* strains, both when compared with free-living bacteria and with endosymbionts in the closely related genus *Wolbachia* (Klasson *et al*. 2009; Weinert *et al*. 2009). *Rickettsia* genomes are also more syntenic than *Wolbachia* genomes (Eremeeva *et al*. 2005; Fenn *et al*. 2006). However, not all *Rickettsia* genomes show these characteristics, and the recently published genomes of *Rickettsia felis* and *Rickettsia bellii* are larger, are less syntenic and have many transposases and proteins with domains involved in protein–protein interactions such as ankyrin repeats and tetratricopeptide repeats (Ogata *et al*. 2005, 2006; Darby *et al*. 2007). The reasons for these qualitative differences in the genome architecture remain unknown, but one possible factor is the presence or absence of conjugation.

Conjugation is an ancient mechanism of horizontal gene transfer that occurs between bacteria through cell contact. This transfer is usually mediated by a plasmid, using a set of conjugation genes (*Tra* genes) that control its regulation, the synthesis of a mating pilus, stabilization contact and DNA metabolism (Clewell 1993). *Rickettsia bellii* has a full complement of these conjugation genes, encoded on its chromosome (Ogata *et al*. 2006). *Rickettsia felis*, on the other hand, has conjugation genes encoded on a plasmid (Ogata *et al*. 2005), and while these appear to be a partial cluster, pili have been observed linking *R. felis* cells, suggesting that conjugation may occur (Ogata *et al*. 2005). Knowledge of conjugation elsewhere in the genus is sketchier. *Rickettsia massiliae*, like *R. bellii,* has a full complement of chromosomal conjugation genes, but this appears to be a very recent transfer event (Blanc *et al*. 2007), and no other complete clusters are known (Eremeeva *et al*. 2005; Blanc *et al*. 2007). Partial *Tra* clusters are known, but are non-functional in some cases (e.g. *Rickettsia monacensis*; Ogata *et al*. 2005; Baldridge *et al*. 2007). Despite these examples, present sampling effort would suggest that conjugation systems are not very common in *Rickettsia*, although there is evidence to suggest that plasmids may be lost through passage in cell culture before genome sequencing (Baldridge *et al*. 2008). As such, the connection, if any, between conjugation and other genomic features remains unclear.

Equally unclear is the broader role of conjugative genes in *Rickettsia* biology. Importantly, however, there is some evidence that they may play a role in pathogenicity. Specifically, the *R. felis* plasmid contains several candidate virulence genes (Ogata *et al*. 2005). In addition, there are two genes that encode proteins with ankyrin repeat domains and seven genes with tetratricopeptide repeat motifs, all of which may be involved in protein–protein interactions (Gillespie *et al*. 2007). Proteins with ankyrin repeat domains are common in eukaryotic chromosomes, but are rare in bacteria. However, they have recently been found in a suite of intracellular bacteria, and some are known to be exported from the bacterial cell, which suggests a role in pathogenicity (Iturbe-Ormaetxe *et al*. 2005; Cho *et al*. 2007; Pan *et al*. 2008).

The aim of this study is to determine the distribution of conjugation genes across *Rickettsia*, as a first step in determining their role in the evolution and biology of the genus. *Rickettsia* are widespread among arthropods, and many of these bacteria branch from near the base of the *Rickettsia* phylogeny (Weinert *et al*. 2009). Based on the phylogenetic analyses of the *R. felis* plasmid genes with homologous genes encoded in *Rickettsia* genomes, it has been suggested that many of the conjugation genes have come from these ‘ancestral’ *Rickettsia* (Gillespie *et al*. 2007). Therefore, we experimentally tested this hypothesis by using PCR to detect the presence of conjugation genes in the new strains of *Rickettsia* we have isolated from arthropods (Weinert *et al*. 2009). In addition, we tested whether conjugation gene phylogenies are decoupled from the bacterial phylogeny, to investigate whether they are being transferred horizontally between bacterial strains.

## 2. Methods

### (a) Identification of conjugation genes

#### (i) Polymerase chain reaction

**Figure 1.**
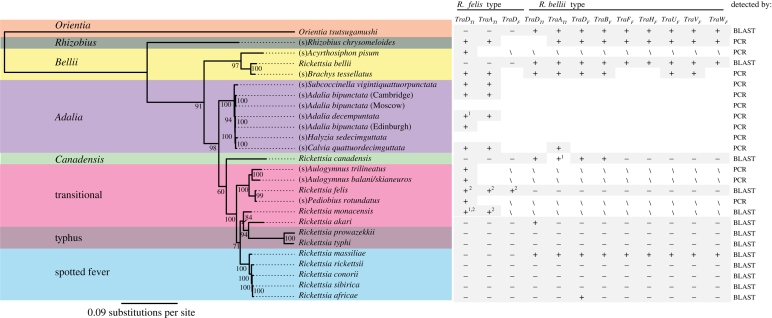
The phylogeny of *Rickettsia* indicating the presence or absence of conjugation genes. *Rickettsia* strains that do not have species names are indicated by the name of their host prefixed by (s). The coloured areas over the phylogenetic tree show the different phylogenetic groups of *Rickettsia*, and posterior support for each clade on the phylogeny is given along the branch length (and is consistent with ML bootstrap support; data not shown). Subscripts of the different conjugation genes indicate either a similarity to the *A. tumificens* plasmid (TI-type) or the *E. coli* F plasmid (F-type). Also indicated is whether genes show closer similarity to the *R. felis* plasmid or the *R. bellii* genome. The genes were detected by either PCR or BLAST searches of published genomes. Four *Rickettsia* strains remain untested for some genes because of a paucity of DNA, and the bellii-type genes of *R. monacensis* remain untested because the genome is unsequenced. +, presence of gene; −, absence of gene; empty, gene not detected by PCR; slash, gene not tested; 1, non-functional; 2, found on plasmid.

Conjugation genes are highly conserved, so we aligned their protein sequences and designed primers in conserved regions to allow their detection by PCR. The conjugation genes in *Rickettsia* are either similar to the *Agrobacterium tumificens* plasmid (TI-type) or the *Escherichia coli* F plasmid (F-type). These two types of plasmids are unrelated and are classified into separate conjugation systems (Garcillán-Barcia *et al*. 2009), so we have added the suffix TI or F to indicate this difference. We tested 13 different strains of *Rickettsia* that were isolated from arthropod hosts (figure 1). Primers were designed using genes from both *R. felis* (where the genes are found on a plasmid) and *R. bellii* (where the genes are chromosomal); these are referred to as felis-type and bellii-type, respectively. In addition, as conjugation genes are usually found in close proximity to each other, primers were designed in some genes to amplify sequences between the genes detected (as gene orientations from unsequenced *Rickettsia* are unknown, primers were designed in all orientations for the *TraD*_*TI*_ and *TraA*_*TI*_ felis-type genes and *TraA*_*TI*_ and *TraD*_*TI*_ bellii-type genes, which were the most common genes detected). All PCR primers and conditions are given in table S1 in the electronic supplementary material. The sequences have been deposited in GenBank under the accession numbers GQ344475–GQ344478 and GQ365372–GQ365409.

#### (ii) BLAST

In order to investigate the presence of conjugation genes in the sequenced *Rickettsia* genomes, we used all of the currently identified conjugation genes from *R. felis* and *R. bellii* to perform BLAST searches. The tblastx program, which translates the query sequence and matches it against a translated database, was used to search for possible homologues in the *Rickettsia* genomes (the genomes and search tool are found at http://patric.vbi.vt.edu/) and in the related genus *Orientia* (http://sourceforge.net/projects/genome-tools/). In addition, two BLAST programs were used to compare the conjugation genes with all nucleotide sequences in GenBank: the computationally intensive tblastx for shorter sequences (less than 300 bp) and the discontiguous megablast algorithm in blastn for larger sequences. Both approaches are designed to pick up less-similar sequences.

### (b) Phylogeny

The phylogeny of the 13 arthropod *Rickettsia*, together with the 11 different *Rickettsia* species whose genomes have been sequenced and a bacterium from the related genus *Orientia*, was reconstructed from four multi-locus strain type (MLST) genes: *atpA*, *coxA*, *gltA* and *16S rDNA* (the sequencing and phylogeny of these genes are described in Weinert *et al*. 2009). The endosymbiont of *Coccidula rufa* was excluded as it is a recombinant strain whose phylogenetic position is uncertain (Weinert *et al*. 2009). Phylogenies of the conjugation genes were constructed for genes that were detected in six or more strains of bacteria. This included the *TraD*_*TI*_, *TraA*_*TI*_ and *TraD*_*F*_ genes, which were similar to those first identified from the *R. felis* plasmid, and *TraA*_*TI*_, *TraD*_*F*_ and *TraB*_*F*_, which were similar to those first identified from the *R. bellii* chromosome. In two cases, the same conjugation genes (*TraD*_*TI*_ and *TraA*_*TI*_) were found on the plasmid of *R. felis* and the chromosome of *R. bellii*, but the gene sequences are too divergent to align accurately. The exception was *TraD*_*F*_ from both types, which could easily be aligned at the protein level and was therefore incorporated into a single gene tree.

The optimal substitution model for each gene was selected using hierarchical likelihood ratio tests in the program Modeltest v. 3.7 (Posada & Crandall 1998). Maximum-likelihood and Bayesian phylogenies were then created, and support for the different nodes were obtained using 1000 bootstrap replicates and posterior probabilities, respectively. Maximum-likelihood phylogenies were built using a maximally parsimonious tree (created using the tree bisection and reconnection branch swapping methods) as a starting tree and then searching tree space using the nearest neighbour interchange branch swapping method in PAUP v. 4b10 (Swofford 2003). Bayesian phylogenies were created using the program MrBayes (Huelsenbeck & Ronquist 2001) to run the MC^3^ algorithm for 1 000 000 generations, with three heated chains and one cold chain, discarding the first 25 per cent of the posterior distribution as burn-in.

### (c) Phylogenetic tests

#### (i) Comparing gene trees with the multi-locus strain-type tree

To test whether the phylogenies of the conjugation genes differed significantly from the MLST tree, we used two different methods. First, taking a Bayesian approach, we calculated the posterior probability that the conjugation gene conformed to the MLST topology (this is estimated by the proportion of the posterior sample of gene trees generated by MrBayes, in which the topology corresponded to the MLST topology). Second, we used a parametric bootstrap or SOWH test of each conjugation gene (Swofford *et al*. 1996; Goldman *et al*. 2000). To carry out these tests, we first calculated the difference between the log likelihood of the maximum-likelihood tree inferred from the conjugation gene sequences (‘ML tree’) and the log likelihood when these sequences were forced to take the topology of the MLST tree (‘null tree’). The observed difference in likelihood was then compared with a distribution of likelihood differences obtained from 1000 datasets simulated under the null hypothesis that the true conjugation gene topology is the MLST tree (Rambaut & Grassly 1997). Each set of simulated sequences was evolved on the topology, branch lengths and model parameters of the null tree. The difference in the log likelihood between the ML and null trees was then recalculated for each of the 1000 simulated datasets. The *p*-value was then calculated as the proportion of the simulated datasets for which the likelihood difference was larger than that of the real data.

#### (ii) Conjugation gene incongruence test

To test whether conjugation genes differed in their evolutionary histories, we also used Bayesian and likelihood-based parametric bootstrap approaches. For the latter, an ML phylogeny was estimated, in which both genes were constrained to the same topology, but each had a unique substitution model and unique branch lengths. The likelihood value was then compared with that obtained when both genes were allowed their own topologies. The observed increase in likelihood was compared with those obtained from 1000 datasets simulated under the null model (different substitution patterns and rates, but a common topology).

#### (iii) Mantel tests

To investigate whether conjugation genes were being preferentially transferred between closely related strains, we tested whether there was a significant correlation in genetic distance between conjugation genes and the MLST genes (Mantel 1967). Pairwise and patristic (branch length) differences were calculated between taxa in the MLST alignment and between taxa in the six different conjugation gene alignments. A correlation coefficient was then calculated between MLST distances and conjugation distances for taxa that were shared between the two phylogenies. Significance of the correlation coefficient was calculated by permuting the conjugation gene taxa names over the MLST taxa names 10 000 times and recalculating the correlation coefficient each time to produce the null distribution. The *p*-value was then obtained using a one-tailed test.

## 3. Results

### (a) Presence of conjugation genes

We used PCR to test a phylogenetically diverse collection of 13 strains of *Rickettsia* for the presence of conjugation genes and combined these results with BLAST searches of the published genomes of *Rickettsia* and the related genus *Orientia*. The pattern of gene presence and absence is summarized in figure 1.

With the PCR approach, 11 of the 13 strains tested positive for at least one conjugation gene, suggesting that conjugation genes are present in most *Rickettsia* lineages (figure 1; PCR products were sequenced in all cases). In one case, the sequence of the conjugation genes contained an internal stop codon, indicating that it no longer encodes a functional protein. We could not detect any conjugation genes in the *Rickettsia* strains found in the ladybird *Adalia bipunctata* from Moscow (strain 10J) or the ladybird *Halyzia sedecimguttata*. However, the lack of a PCR product is not confirmation that the genes are not present, as they might not have been amplified if the genes are too divergent for the primers to anneal or if they have been truncated (as is common in *Rickettsia* conjugation genes). In addition to these findings, we also detected polymorphism in the sequencing reads at some sites in some genes of the symbionts of *Rhizobius chrysomeloides*, *Brachys tessellatus, C. rufa*, *Adalia decempunctata* and *Calvia quattuordecimguttata*, which indicate duplication events or multiple plasmids. However, this does not impact our phylogenetic analysis as the divergence between the copies was very small compared with the divergence between conjugation genes of different strains. The BLAST searches of *Rickettsia* genomes confirmed that only five of the eleven named species contained conjugation genes. A list of gene identifiers of the best BLAST hits used in constructing conjugation gene phylogenies is given in table S2 in the electronic supplementary material. The genomes that lack these genes fall into a closely related cluster of vertebrate pathogens, so it appears that the ancestral *Rickettsia* probably possessed conjugation genes, implying that they have only recently been lost from many of the vertebrate pathogens (figure 1).

Figure 1 also reveals patterns of co-occurrence between the genes that are close in sequence to the *R. felis* plasmid (felis-type) and those that are closer to the *R. bellii* genome (bellii-type), which have not yet been found on plasmids. It is clear that genes of both felis- and bellii-type tend to co-occur, with most strains having either several genes of a particular type or none at all (figure 1). Genes of both types also occur in all four of the main *Rickettsia* clades studied, and bellii*-*type genes are also found in the closely related *Orientia tsutsugamushi*.

### (b) Independent introductions of TI-type conjugation genes into *Rickettsia*

BLAST searches of the conjugation genes across all sequences in GenBank also revealed differences in the evolutionary histories of the genes from the two separate conjugation systems: F-type and TI-type. F-type conjugation genes appear to constitute a *Rickettsia*-specific clade, with *TraB*_*F*_ and *TraD*_*F*_ (from both *R. felis* and *R. bellii*) giving best hits to all other *Rickettsia* sequences. In contrast, the TI-type conjugation genes seemed to have originated from two independent transfers into *Rickettsia*. A tblastx search of the *TraA*_*TI*_ gene from *R. bellii* indicated that this gene is more similar to *TraA*_*TI*_ genes from numerous other bacteria than it is to the *TraA*_*TI*_ gene from the *R. felis* plasmid, with the reciprocal tblastx giving a similar result. The *TraD*_*TI*_ gene from *R. bellii* also gave a higher similarity to other bacteria, although the reciprocal tblastx query gave a significant hit only to the *TraD*_*TI*_ genes of various *Rickettsia* species. Phylogenies based on the tblastx output showing evidence of separate origins for the *TraA*_*TI*_ and *TraD*_*TI*_ genes are given in fig. S1 in the electronic supplementary material.

### (c) Synteny of conjugation genes is broken

**Figure 2.**
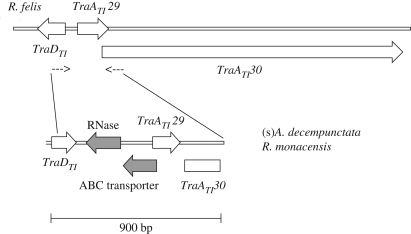
Lack of synteny in the conjugation genes in *R. felis* compared with *R. monacensis* and the symbiont of *A. decempunctata*. Small arrows represent the position of the PCR primers.

To investigate whether conjugation genes are syntenic in different species, we attempted to amplify the region between some of the genes (see §2). All attempts but one failed, implying that these conjugation genes are not in the same orientation as they are found in on the *R. felis* plasmid or on the *R. bellii* chromosome. The only successful PCR amplified the region between the felis-type genes *TraA*_*TI*_ and *TraD*_*TI*_ from the endosymbiont of *A. decempunctata* and gave a product that was larger than expected. Sequencing showed that, relative to the *R. felis* plasmid, two genes were inserted between the conjugation genes, and the *TraD*_*TI*_ gene was in the opposite orientation; this is illustrated in figure 2. In addition, this four-gene fragment of (s)*A. decempunctata* gave a blastn hit to the nucleotide sequence from the *R. monacensis* plasmid, indicating the same pattern in *R. monacensis*. This is surprising given that *R. monacensis* is more closely related to *R. felis* than to the symbiont of *A. decempunctata*.

### (d) Phylogenetic incongruence of multi-locus strain type and conjugation genes

**Figure 3.**
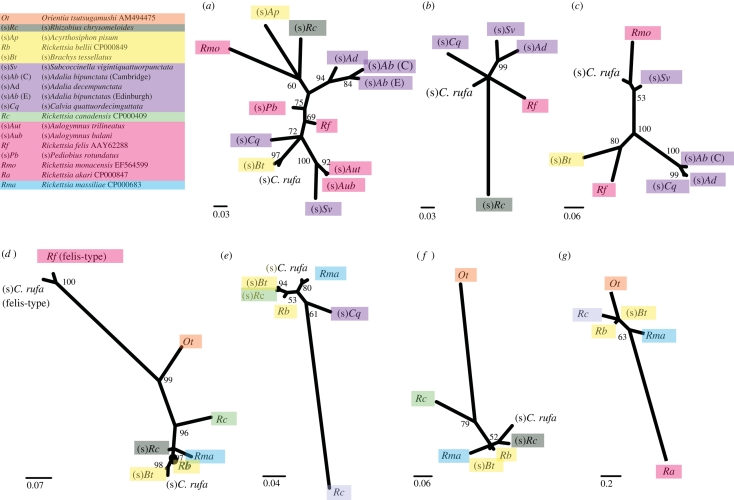
Phylogeny of conjugation genes detected in six or more strains of *Rickettsia* (*a*) *TraD*_*TI*_ (felis-type), (*b*) *TraA*_*TI*_3′ (felis-type), (*c*) *TraA*_*TI*_5′ (felis-type), (*d*) *TraD*_*F*_ (bellii- and felis-type), (*e*) *TraA*_*TI*_ (bellii-type), (*f*) *TraB*_*F*_ (bellii-type) and (*g*) *TraD*_*TI*_ (bellii-type). The felis-type *TraA*_*TI*_ gene is considerably larger than other conjugation genes (figure 2), and we sequenced two fragments separately. However, as incongruence was confirmed by a parametric bootstrap test (see §2; probability of a common topology: *p* = 0.002), the phylogenies are depicted separately in (*b*) and (*c*). Scale bars represent the number of substitutions per site. Posterior support is given along the branches (and is consistent with ML bootstrap support; data not shown). Coloured boxes represent the different phylogenetic groups of *Rickettsia*, as shown in figure 1.

To test whether conjugation genes are horizontally transmitted between *Rickettsia* strains, we reconstructed maximum-likelihood and Bayesian phylogenies of conjugation genes that were detected in six or more strains of bacteria. These included *TraD*_*TI*_ and *TraA*_*TI*_ of felis-type and *TraD*_*TI*_, *TraD*_*F*_, *TraA*_*TI*_ and *TraB*_*F*_ of bellii-type and are depicted in figure 3. The two genes *TraD*_*TI*_ and *TraA*_*TI*_ were found in both the *R. felis* and *R. bellii* clusters, but as the BLAST searches suggested independent introductions of TI-type genes into *Rickettsia*, we analysed them separately (they are, in any case, too divergent to align with confidence). In contrast, the two felis-type *TraD*_*F*_ were combined with the bellii-type genes into a single phylogeny (figure 3*d*).

#### (i) Phylogenetic tests

We used two tests to investigate whether the phylogenies of the conjugation genes differed significantly from the phylogeny of the bacteria (reconstructed from the MLST genes). For our Bayesian trees, we calculated the posterior probability that each gene topology corresponded to the MLST topology. For our maximum-likelihood trees, we carried out SOWH tests. Results, in table 1, show that in all cases the phylogenies of the conjugation genes differed significantly from those of the four MLST genes. This strongly suggests extensive horizontal gene transfer of conjugation genes in *Rickettsia*.

**Table 1.** Bayesian and SOWH tests for incongruence between the bacterial MLST phylogeny (figure 1) and the individual conjugation gene trees (figure 3); and Mantel tests for a correlation between the genetic distances of the conjugation genes and other genes (see text for full details). Also shown are the best-fit models of sequence evolution: TrN + *Γ*_5_ (Tamura–Nei with a five-category discretized gamma distribution of rates across sites) and HKY + *Γ*_5_ (Hasegawa, Kishino and Yano with a gamma distribution; Posada & Crandall 1998). The Mantel test on pairwise differences is absent for the gene *TraB*_*F*_ because the *Rickettsia canadensis* gene is truncated and does not overlap with the sequence from some of the other symbionts. *p < 0.05; **p < 0.07; ***p < 0.001.

		SOWH test				Bayesian test	Mantel test			
							patristic distance		pairwise distance	
gene (type)	model	−ln L(ML)	−ln L(MLST)	Δln L	*p*-value	*p*-value	*r-*value	*p*-value	*r-*value	*p*-value
*TraD*_*TI*_ (felis)	TrN+*Γ*_5_	647.081	659.118	12.04	<0.001***	<0.001***	0.236	0.15	0.940	0.083
*TraA*_*TI*_ 3′ (felis)	HKY+*Γ*_5_	3249.779	3255.671	5.892	0.001***	0.002**	0.935	0.075	0.265	0.168
*TraA*_*TI*_ 5′ (felis)	HKY+*Γ*_5_	766.426	814.515	48.09	<0.001***	<0.001***	0.436	0.053	−0.069	0.232
*TraD*_*F*_ (bellii and felis)	HKY+*Γ*_5_	637.756	645.868	8.112	<0.001***	<0.001***	0.102	0.235	0.018	0.349
*TraA*_*TI*_ (bellii)	HKY+*Γ*_5_	848.214	851.482	3.267	0.009**	0.002**	−0.083	0.319	n/a	n/a
*TraB*_*F*_ (bellii)	HKY+*Γ*_5_	409.635	414.126	4.491	0.006**	0.014*	0.907	0.042*	0.183	0.106
*TraD*_*TI*_ (bellii)	HKY+*Γ*_5_	848.903	851.755	2.852	0.046*	0.019*	−0.189	0.342	0.052	0.249

### (e) No evidence that conjugation genes co-speciate with the bacteria or preferentially move between closely related bacterial strains

While it is apparent that the conjugation gene phylogenies are extensively decoupled from the MLST phylogeny, there also seems to be a few cases in which closely related strains of *Rickettsia* have related conjugation genes. For example, on the *TraD*_*TI*_ (felis-type) phylogeny (figure 3*a*), symbionts of the parasitoid wasp genus *Aulogymnus* group together with strong posterior support (93%), which is also the case on the bacterial phylogeny (figure 1). These associations could indicate vertical transmission of conjugation genes, or that conjugation genes move preferentially between related strains of bacteria. However, the associations might also have arisen by chance. To distinguish between these possibilities, we used Mantel tests of independence between the MLST and conjugation gene trees (Mantel 1967; see §2). Table 1 shows that the tests in some cases reach marginal significance, but when multiple testing is considered, the degree of association appears no larger than would be expected by chance.

### (f) Patterns of horizontal transfer

Horizontal transfer of conjugation genes seems to occur more often between the major groups than within the major groups. Figure 3 shows the phylogeny of the conjugation genes coloured as in figure 1 according to the group of *Rickettsia* host from which they were isolated (major groups defined by Weinert *et al*. 2009). Although numerous examples exist of between-group transfer where groups are not monophyletic (e.g. in figure 3*a*,*c,e*) and have a different order of divergence (e.g. in figure 3*b*,*d,f*), there is evidence for only two cases of horizontal transfer within the adalia group in figure 3*a,b*.

## 4. Discussion

We have found that conjugation genes are common in the genus *Rickettsia*, including those strains thought to exclusively infect arthropods, which represent the majority of the group (Weinert *et al*. 2009). Most of the strains tested here are positive for the *R. felis* plasmid-type genes (figure 1), and since this class of conjugation genes has so far only been discovered on a plasmid (Ogata *et al*. 2005; Baldridge *et al*. 2007), this suggests that plasmids may be common in the genus. However, further experiments will be needed to establish the physical position of these elements. These results are in contrast to other obligate intracellular pathogens, only a handful of which contain conjugative plasmids (Samuel *et al*. 1983; Stephens *et al*. 1998; Ogata *et al*. 2005; Gillespie *et al*. 2007).

We have also shown that conjugation genes within *Rickettsia* undergo horizontal transfer at a high rate. This is in stark contrast to the rest of genome, in which horizontal transfer is rare (Weinert *et al*. 2009). Furthermore, most cases of movement of conjugation genes are between different major groups, and there is no evidence for any correspondence between the bacterial phylogeny and the conjugation gene phylogeny (table 1). Therefore, there is no evidence that the conjugation genes preferentially move between closely related strains or co-speciate with the bacteria. This is surprising, given that related strains of *Rickettsia* tend to infect related arthropod hosts and suggest a high rate of DNA transfer over evolutionary time.

We have further found evidence of two separate evolutionary origins of conjugation genes in *Rickettsia*. The F-type conjugation genes that we identified appeared to constitute a *Rickettsia*-specific clade, and the significant divergence between copies of these genes suggests that they have been evolving in *Rickettsia* for some time. This raises the question as to why these genes have been maintained in *Rickettsia*. It is possible that some plasmids exist as entirely selfish elements, but this would require a high degree of infectious transmission (Paulsson 2002), which seems unlikely given the intracellular lifestyle of *Rickettsia*. The *Rickettsia* strains we have studied probably only infect arthropods and probably have a biology similar to the related genus *Wolbachia. Wolbachia* also has an extremely high rate of horizontal gene transfer, thought to be driven by a phage (Klasson *et al*. 2009). Also, in *Wolbachia*, mobile genetic elements are associated with differences in the way in which the bacteria manipulate the reproduction of the host insect (Sinkins *et al*. 2005), and it is therefore tempting to speculate that conjugative elements may play a role in the exchanging genes that control the reproductive manipulation of *Rickettsia* hosts. Although this is purely speculative, the lack of these genes in strains that infect vertebrates argues against a role in vertebrate pathogenicity. Indeed, unlike many other bacteria, horizontally transferred elements in *Rickettsia* are not associated with vertebrate pathogenicity (Darby *et al*. 2007).

The presence of a system that can horizontally transfer DNA between *Rickettsia* species is likely to have an important effect on the evolution of these strains. The intracellular lifestyle and maternal transmission of these bacteria reduce their effective population size and increase their mutation rate. Consequently, endosymbionts suffer the accumulation of deleterious mutations owing to Muller's ratchet and interference selection (Moran 1996). Horizontal gene transfer can counteract these problems by allowing deleterious mutations to be purged from the population. Indeed, there seems to be evidence of ‘regenerative’ DNA exchange in *Neisseria* (Treangen *et al*. 2008). However, if most of the genome is not experiencing recombination (Weinert *et al*. 2009), this argues against conjugation genes affecting *Rickettsia* in this particular way.

Processes such as Muller's ratchet have led to small genomes of largely essential genes, and this may create another problem, as these genomes may have limited potential to evolve novel traits. This is likely to be a particular problem for *Rickettsia*, as most strains are probably parasites and will therefore face strong selection pressures from host resistance. Horizontal gene transfer can counteract these problems by providing novel genes or combinations of genes. Indeed, in other bacteria, genes involved in pathogenicity are often carried on mobile genetic elements such as plasmids. Therefore, a conjugative system that horizontally transfers DNA in *Rickettsia* suggests a higher probability of acquiring novel traits, which may facilitate epidemic outbreaks, given that *Rickettsia* are classified as potential emerging pathogens (Walker & Ismail 2008). However, many of the vertebrate pathogenic *Rickettsia* lack conjugation genes, which suggests that, in these cases, conjugation genes are not important in vertebrate pathogenicity.

If plasmids are widespread among arthropod *Rickettsia*, they could be exploited in a range of applications. For example, it has been suggested that bacterial endosymbionts could be used to drive genes through insect populations in order to block the transmission of disease (Sinkins & Gould 2006). However, this has been hampered by the difficulty of transforming symbionts such as *Wolbachia*. It is possible that *Rickettsia* may be a more promising system to use in the genetic manipulation of populations. Although transposon-based mutagenesis has seen significant recent advances in *Rickettsia* biology (Liu *et al*. 2007) and greatly facilitates functional genetic analysis, no system currently exists to deliver foreign genes into *Rickettsia*, so a natural genetic transformation tool is a distinct advantage.

In conclusion, the presence of plasmids in *Rickettsia* is an exciting discovery that is likely to reveal insights into the biology of the different strains and may be a useful tool in the genetic manipulation of intracellular bacteria.
